# Electrodeposited Sulfur and Co_x_S Electrocatalyst on Buckypaper as High-Performance Cathode for Li–S Batteries

**DOI:** 10.1007/s40820-020-00479-1

**Published:** 2020-07-03

**Authors:** Yi Zhan, Andrea Buffa, Linghui Yu, Zhichuan J. Xu, Daniel Mandler

**Affiliations:** 1 School of Materials Science and Engineering, Nanyang Technological University, Singapore, 639798 Singapore; 2 Institute of Chemistry, The Hebrew University of Jerusalem, 9190401 Jerusalem, Israel; 3 Campus for Research Excellence and Technological Enterprise (CREATE), Singapore-HUJ Alliance for Research and Enterprise (SHARE), Singapore, 138602 Singapore

**Keywords:** Electrodeposition, Lithium sulfur batteries, Buckypaper, Electrocatalysts

## Abstract

**Abstract:**

Lithium–sulfur batteries (LSBs) are considered as the next generation of advanced rechargeable batteries because of their high energy density. In this study, sulfur and Co_x_S electrocatalyst are deposited on carbon nanotube buckypaper (S/Co_x_S/BP) by a facile electrodeposition method and are used as a binder-free high-performance cathode for LSBs. Elemental sulfur is deposited on buckypaper by electrooxidation of a polysulfide solution (~ S_6_^2−^). This approach substantially increased the current and time efficiency of sulfur electrochemical deposition on conductive material for LSBs. S/Co_x_S/BP cathode could deliver an initial discharge capacity as high as 1650 mAh g^−1^ at 0.1 C, which is close to the theoretical capacity of sulfur. At current rate of 0.5 C, the S/Co_x_S/BP has a capacity of 1420 mAh g^−1^ at the first cycle and 715 mAh g^−1^ after 500 cycles with a fading rate of 0.099% per cycle. The high capacity of S/Co_x_S/BP is attributed to both the homogeneous dispersion of nanosized sulfur within BP and the presence of Co_x_S catalyst. The sodium dodecyl sulfate (SDS) pretreatment of BP renders it polarity to bind polysulfides and thus facilitates the good dispersibility of nanosized sulfur within BP. Co_x_S catalyst accelerates the kinetics of polysulfide conversion and reduces the presence of polysulfide in the cathode, which suppresses the polysulfide diffusion to anode, i.e., the shuttle effect. The mitigation of the active material loss improves not only the capacity but also the cyclability of S/Co_x_S/BP.

**Graphic Abstract:**

**Electronic supplementary material:**

The online version of this article (10.1007/s40820-020-00479-1) contains supplementary material, which is available to authorized users.

## Highlights

- Nanosized sulfur and Co_x_S electrocatalyst are electrodeposited on carbon nanotube buckypaper (S/Co_x_S/BP) as a binder-free high-performance cathode for lithium–sulfur batteries (LSBs).
- Both electrooxidation of a polysulfide solution (~S_6_^2−^) to sulfur and the electrodeposition of highly active Co_x_S catalyst substantially increase the current and time efficiency of cathode preparation for LSBs.

**Electronic supplementary material:**

The online version of this article (10.1007/s40820-020-00479-1) contains supplementary material, which is available to authorized users.

## Graphical abstract

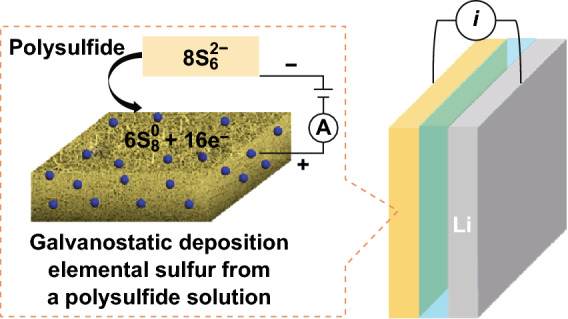

## Introduction

Lithium-ion batteries have achieved enormous success in a variety of applications such as consumer electronics, hybrid electric vehicles and electric vehicles. Yet, the increasing demand for energy storage systems with higher energy density has promoted the search for the next generation of advanced rechargeable batteries [1–3]. Lithium–sulfur batteries (LSBs) are among the promising candidates, because sulfur, the active material in the cathode, is not only low cost, earth abundant and environment benign, but also possesses a theoretical capacity as high as 1675 mAh g^−1^ by hosting two Li^+^ per sulfur atom [4]. However, LSBs suffer from several technical challenges, especially on the sulfur cathode [5].

Both sulfur and its discharge products (Li_2_S_2_ and Li_2_S) are not conductive, leading to poor electron transfer and as such sluggish kinetics [1, 6]. Suffering from a large volume expansion of nearly 80% after discharging, sulfur also bears the pulverization and the loss of sulfur active material from the cathode, which is a major cause of capacity decay. Impregnating sulfur into porous nanostructured carbonaceous materials is a common strategy to increase the conductivity and mitigate the volume expansion of sulfur [7–9]. Although achieving exciting progress, such strategy also brings the challenge of the low ratio (< 70 wt%) of active sulfur in the carbon–sulfur composite. The carbon hosts with porous structures are usually fabricated by complex processes, and their structures and surface chemistry properties cannot ensure the complete and homogeneous sulfur distribution after sulfur impregnation. Moreover, the sulfur ratio is further decreased to ~ 50 wt% considering the addition of polymer binders and conductive agents in the conventional cathode fabrication using a slurry coating method. The non-active materials not only reduce the capacity based on the total mass of the electrode but also increase the electrode polarization, which is especially prominent at high current density. With the advantages of simplicity, low cost and high controllability, electrodeposition can be an appealing alternative to fabricate binder-free cathode with 100 wt% sulfur on current collector and rule out the negative effects of non-active additives. The naturally electrical connection of electrodeposited sulfur with the substrate also ensures the high utilization of active sulfur (nearly 100% in theory). Only a few reports have utilized sulfur electrodeposition for cathode fabrication in LSBs [10–12]. However, the electrodeposition was usually time-consuming due to the formation of polysulfides in the sulfide precursor solution.

Furthermore, while sulfur and the final discharge products are insoluble in most electrolytes, the intermediate products, i.e., polysulfides, are highly soluble in the battery electrolyte. During the charge–discharge process, polysulfides can diffuse to the anode side and react directly with metallic lithium. This results in the irreversible loss of active material and thus causes the rapid capacity decay during cycling, which is known as the shuttle effect. It is well known that polar polysulfides can be well adsorbed by polar host materials via polar–polar interactions [13]. A wide variety of such polar hosts have been developed to mitigate the shuttle effect of polysulfides at the cathode, including modified carbonaceous materials, functional polymeric materials, metal oxides and metal sulfides [13–18]. Recent research also found a catalytic effect on polysulfide conversion by several materials such as cobalt, platinum, MoS_2-x_ and ZnS, thereby promoting the polysulfide conversion and reducing the presence of polysulfides in the electrolyte and suppressing the shuttle effect [19–23].

Herein, a binder-free electrode, consisting of both sulfur active material and Co_x_S catalyst electrodeposited on buckypaper (S/Co_x_S/BP), was developed as a high-performance cathode for LSBs. Polysulfide solution instead of sulfide solution was used in the electrodeposition, which largely shortens the deposition time from hours to minutes. BP acted as substrate with high surface area and good electrical conductivity. The S/BP cathode delivered an initial discharge capacity as high as 1400 mAh g^−1^ at 0.1 C and showed good cycling stability with a decay rate of 0.16% per cycle for 170 cycles at 0.5 C. Acting as both the polar host material and the catalyst of polysulfide conversion, the presence of Co_x_S promoted the S/Co_x_S/BP cathode to deliver an initial discharge capacity as high as 1650 mAh g^−1^ at 0.1 C and showed good cycling stability with a decay rate of 0.099% per cycle for 500 cycles at 0.5 C.

## Experimental

### Chemicals

All chemicals were used as received. Cobalt nitrate hexahydrate (Co(NO_3_)_2_·6H_2_O, 99.99%), thiourea (H_2_NCSNH_2_, 99%), sodium dodecyl sulfate (CH_3_(CH_2_)_11_OSO_3_Na, 99%), sodium hydrosulfide hydrate (NaHS·xH_2_O, NaHS ≥ 60%), sodium hydroxide (NaOH, 98%), 1,3-dioxolane (DOL, 99.8 wt%), 1,2-dimethoxyethane (DME, 99.5 wt%), lithium bis(trifluoromethanesulfonyl) imide (LiTFSI, 99.95 wt%), lithium nitrate (LiNO_3_, 99.99%), lithium sulfide (Li_2_S, 99.98%) and sulfur (S, flakes, 99.99%) were purchased from Sigma-Aldrich. Ultrapure water (UPW) filtered by a Millipore Milli-Q Integral Water Purification System (Millipore Corp.) was used as the common solvent.

### Synthesis

#### Electrodeposition of Sulfur

Polysulfide solution was prepared by dissolving 50 mmol NaHS·xH_2_O, 90 mmol NaOH and 250 mmol sulfur in 50 mL UPW at 80 °C overnight to obtain 1 M ~ S_6_^2−^ solution. The electrodeposition of sulfur on buckypaper (BP, NanoTechLabs, 60 g m^−2^, ~ 250 µm in thickness) was carried out in a three-electrode compartment at ambient conditions by a galvanostatic method with current density of 10 mA cm^−2^ using a Solartron potentiostat (Solartron 1470E). BP (1 × 1.5 cm^2^) was first wetted to improve the accessibility of polysulfide by immersing the area of 1 × 1 cm^2^ in 0.1 wt% sodium dodecyl sulfate (SDS) solution. Then, it was directly used as the working electrode in the polysulfide solution using a Pt foil (1.5 × 1.5 cm^2^) and an Ag/AgCl (in 3 M KCl) electrode as the counterelectrode and reference electrode, respectively. Sulfur was electrochemically deposited on BP, which was pretreated by different concentrations of SDS solutions (0.05–1 wt%), under constant current density (2–50 mA cm^−2^). After deposition, S/BP was carefully rinsed for three times by copious UPW and finally dried overnight in an oven at 60 °C. Several experiments were conducted with ethanol instead of SDS solution as the wetting agent and denoted as S/BP-EtOH.

#### ***Preparation of Co***_***x***_***S-decorated BP (Co***_***x***_***S/BP) and its Further Sulfur Deposition (S/Co***_***x***_***S/BP)***

**Scheme 1:**
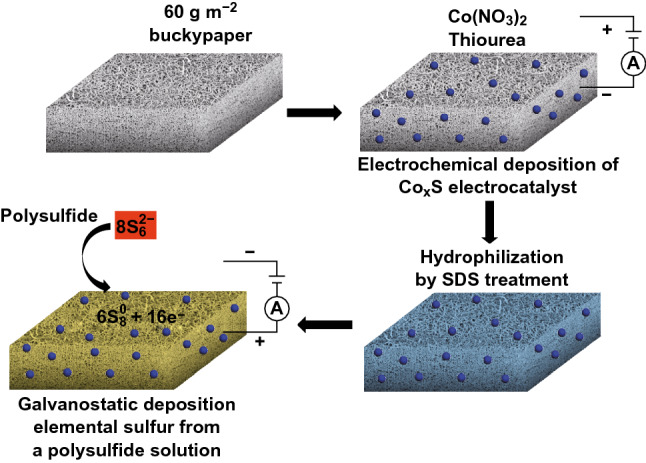
S/Co_x_S/BP electrode preparation

The electrodeposition of Co_x_S on BP was carried out in a two-electrode compartment at ambient conditions by applying a constant voltage (MINI PRO 300 V power supply, Major Science). BP (1 × 1.5 cm^2^) was used as the cathode by immersing an area of 1 × 1 cm^2^ into the isopropanol solution containing 100 mM H_2_NCSNH_2_ and 5 mM Co(NO_3_)_2_. Pt foil (1.5 × 1.5 cm^2^) was used as the anode. The applied voltage was 20 V, and the time was 1 min. The Co_x_S/BP was washed with ethanol and UPW for several times and dried overnight at 60 °C. The loading of Co_x_S was ~ 0.2 mg cm^−2^ determined by the weight difference of BP before and after electrodeposition. Co_x_S/BP was further annealed at 300 or 600 °C (samples denoted as Co_x_S300/BP and Co_x_S600/BP, respectively) for 2 h in Ar atmosphere to study the annealing effect. Co_x_S was also similarly prepared on 304 stainless steel with 270 mesh to rule out the catalytic effect of BP. Sulfur was also electrodeposited on Co_x_S/BP, Co_x_S300/BP and Co_x_S600/BP, and the samples were denoted as S/Co_x_S/BP, S/Co_x_S300/BP and S/Co_x_S600/BP, respectively. The complete fabrication process of the S/CoxS/BP cathode is described in Scheme 1.

### Morphology and Structure Characterization

Field emission scanning electron microscopy (SEM) was performed on a Zeiss Supra 55 microscope operating at 5 kV accelerating voltage. Field emission transmission electron microscopy (TEM) was carried out on a JOEL 2010 microscope operating at 200 kV accelerating voltage. X-ray diffraction (XRD) patterns were obtained by a Bruker GADDS XRD powder diffractometer using a Cu Kα source (*λ* = 1.5418 Å) at 40 kV and 30 mA. X-ray photoelectron spectroscopy (XPS) analysis was performed on a Kratos Axis Supra with delay-line detector spectrometer. Energy-dispersive X-ray spectroscopy (EDS) was performed by X Max detector, Oxford Instruments, UK, and used for elemental mapping.

### Adsorption Test of Polysulfides, Cell Assembly and Electrochemical Measurements

1 mM Li_2_S_6_ solution was prepared by dissolving Li_2_S and sulfur with the corresponding stoichiometry in the 1:1 (v/v) DOL/DME solute. The adsorption test was carried out by adding 1 mg BP, Co_x_S, Co_x_S300 or Co_x_S600 in 1 mL each of the polysulfide solution.

Symmetric electrochemical cells were assembled by the following procedure: CR2032 coin cells were assembled in an Ar-filled glove box by using two identical 1 × 1 cm^2^ electrodes (Co_x_S/BP, BP and Co_x_S; Co_x_S loading is ca. 0.2–0.3 mg cm^−2^) as cathode and anode, a Celgard 2325 separator and 30 µL electrolyte of 1 M LiTFSI and 0.1 M Li_2_S_6_ in a 1:1 (v/v) DOL/DME mixture. The working electrode after the test was disassembled from the cell, rinsed with DOL thrice to remove the lithium salt on the surface and then evacuated overnight at room temperature for ex situ analysis on the next day.

Li–S batteries were assembled by the direct use of S/BP or S/Co_x_S/BP (1.0–1.5 mg cm^−2^ of sulfur loading on 1 × 1 cm^2^ BP of ca. 6 mg) as cathode and Li-metal foil (China Energy Lithium Co., China; 16 mm in diameter and 0.6 mm in thickness, ca. 64 mg) as anode in CR2032 type coin cells with a Celgard 2325 separator and 30 µL electrolyte of 1 M LiTFSI in DOL/DME (1:1 v/v) with 1 wt% LiNO_3_. The sulfur content was ca. 14.3 ~ 20 wt% in the cathode, and E/S ratio was 20 ~ 30 μL mg _S_^−1^. Cyclic voltammetry (CV) measurements were carried out using a Solartron 1470E potentiostat. The galvanostatic charge–discharge cycles were performed on a Neware battery tester at the ambient conditions. The cathode specific capacities were normalized by the sulfur mass.

## Results and Discussion

### Electrodeposition of Sulfur on Buckypaper (S/BP)

Polysulfide solution was prepared and used as the electrodeposition solution instead of sulfide solution, which is commonly employed in the literature [10, 12]. The oxidation of sulfide to sulfur is a multi-step process: sulfide is oxidized to form the short-chain polysulfides first and then the long-chain polysulfides, and the latter are finally oxidized to sulfur. The overall reactions can be expressed as follows:

1 $$mS^{2 - } \to S_{m}^{2 - } + \left( {2m - 2} \right)e^{ - } \left( {m = 2\sim 4} \right)$$

2 $$nS_{m}^{2 - } \to mS_{n}^{2 - } + \left( {2n - 2m} \right)e^{ - } \left( {n = 5\sim 7} \right)$$

3 $$8S_{n}^{2 - } \to nS_{8} + 16e^{ - }$$

**Fig. 1:**
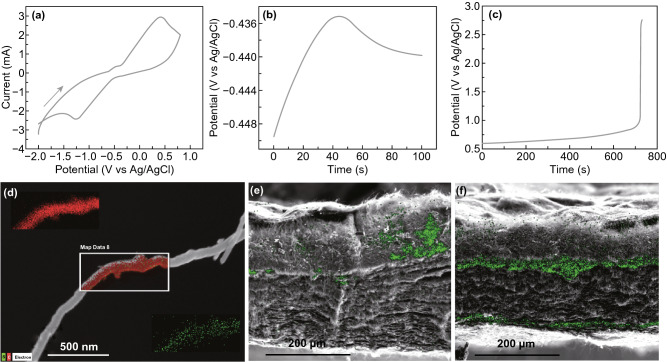
**a** CV of 10 mM polysulfide using a BP electrode at 20 mV s^−1^ scan rate. Chronopotentiometry of sulfur electrodeposition on BP electrode at **b** 10 mA cm^−2^ and **c** 35 mA cm^−2^. **d** EDS mapping of sulfur (green) and carbon (red) of a single nanotube of S/BP. Overlay of EDS sulfur mapping on the SEM image of the cross section of **e** S/BP-EtOH and **f** S/BP. The upper side of the BP corresponds to the side oriented to the counterelectrode during deposition

Therefore, the direct oxidation of long-chain polysulfides will definitely shorten the electrodeposition process and as such save time. Sulfur electrodeposition was carried out galvanostatically (constant current) using BP as the working electrode, Pt foil as the counterelectrode and Ag/AgCl as the reference electrode. The hydrophobicity makes BP difficult to wet in the polysulfide solution. Therefore, a pretreatment of the PB was crucial for the efficient electrochemical deposition of sulfur. A solution of 0.1 wt% SDS was used to wet the BP surface. We anticipated that the amphiphilic SDS will, on the one hand, adsorb on the carbon nanotubes and, on the other hand, form a polar sulfate layer, which could interact with the polar polysulfides. Such an approach has been researched by Chen et al. [24]. The accessibility of the polysulfide ions to the highly porous BP is expected to be driven by the electric field and capillary forces. On the BP surface, polysulfide undergoes 2e^−^ oxidation and deposits as elemental sulfur. Electrodeposition lasted several minutes (Fig. 1c) while it took up to 4 h in a previous study to reach similar loading using sulfide (instead of a polysulfide) solution [12].

The facile elemental sulfur electrodeposition is confirmed by the CV of polysulfide using a BP electrode (Fig. 1a). The CV shows the quasi-reversible electrochemical responses of polysulfide oxidation and sulfur reduction that occur at a small overpotential as compared to standard reduction potential for this reaction of −0.34 V.

The chronopotentiometry of sulfur deposition on BP (Fig. 1b) shows an initial increase in the overpotential required for polysulfide oxidation, followed by a decrease at ca. 50 s of deposition. This behavior can be explained as the initial formation of sulfur nuclei and their consecutive growth with increase in the surface area. According to our experiments, galvanostatic sulfur deposition could be carried on until complete saturation of the BP, which results in an abrupt increase in the electrical resistance and deposition potential (Fig. 1c). The following cell performance results will show that over amount of sulfur is detrimental to the battery capacity and cycling performance; however, electrochemical deposition enables fine-tuning of the sulfur density for the optimization of the battery performance.

EDS sulfur mapping performed on a single carbon nanotube (Fig. 1d) taken from a S/BP electrode shows that sulfur is deposited as a thin layer on the surface of the carbon nanotube and it does not simply occupy the pores of BP. This ensures that all the sulfur is electrically connected to the BP and available for the electrochemical reaction. Sulfur mapping of the S/BP and S/BP-EtOH cross sections was performed to verify sulfur distribution and differences between the two treatments. S/BP-EtOH cross section (Fig. 1e) shows that sulfur is unevenly distributed, and it accumulates mostly on the side of the BP oriented toward the counterelectrode during deposition. On the other hand, sulfur mapping of the S/BP cross section (Fig. 1f) shows that sulfur is evenly distributed horizontally on two layers parallel to the faces of the electrode. Yet, also in S/BP, polysulfide fails to penetrate deeply into BP, resulting in only near-surface deposition of sulfur but void deposition in the bulk. Surface elemental mapping demonstrates quite homogeneous dispersion of sulfur nanoparticles on CNTs of BP surface (Fig. S1).

**Fig. 2:**
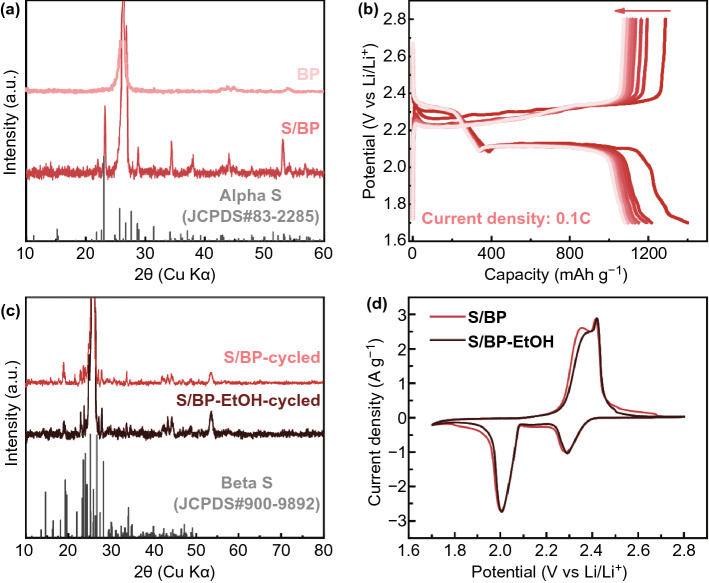
**a** XRD pattern of S/BP. **b** Discharge–charge profile of S/BP at a rate of 0.1 C. **c** XRD patterns of S/BP and S/BP-EtOH after one cycle of charge–discharge. **d** CV of S/BP and S/BP-EtOH at 0.1 mV s^−1^

XRD pattern (Fig. 2a) shows that all other peaks of S/BP, besides those attributed to BP, correspond well with the standard file of orthorhombic α-sulfur (JCPDS No. 83-2285), suggesting the successful sulfur electrodeposition on BP. The sulfur phase was the same as that reported in previous studies [10–12]. In contrast, the sulfur electrodeposited on BP treated by ethanol (S/BP-EtOH) instead of SDS solution (Fig. S2a) was indexed as monoclinic β-sulfur. Ethanol presumably fully wetted the hydrophobic BP due to its low surface tension. However, unlike SDS forming a polar layer on carbon nanotubes (CNTs) surface in BP, CNTs surface treated by ethanol was still nonpolar and had poor affinity to polysulfide, and thus, sulfur particles electrodeposited on BP-EtOH have the tendency to aggregate.

The charge–discharge profile at a current rate of 0.1 C showed two discharge plateaus and the corresponding charge plateaus with close spacing (Fig. 2b). The two discharge plateaus were related with the sulfur reduction to long-chain polysulfides and then to short-chain polysulfides. The charge plateaus represent the reverse process. S/BP could deliver as high capacity as 1400 mAh g^−1^ while S/BP-EtOH had a capacity of ca. 1197 mAh g^−1^ at 0.1 C (1 C = 1600 mAh g^−1^), indicating the superior capacity of S/BP to that of S/BP-EtOH (Fig. S2b). Both of their capacities became relatively stable after 10 cycles. The effects of SDS concentration and deposition current density were studied on the S/BP performance in this work. Cycling performance was compared among S/BP electrodes prepared by different SDS concentrations and by different current densities. The initial capacities were in the range of 1200 ~ 1270 mAh g^−1^ and the final capacity after 300 cycles for the S/BP were ca. 790 ~ 860 mAh g^−1^, indicating that the effect of the electrodeposition current densities on the S/BP performances was small (Fig. S2c). This was also the case for S/BP pretreated by different SDS concentrations (Fig. S2d).

Interestingly, both S/BP and S/BP-EtOH were indexed as β-sulfur after only one charge–discharge cycle, suggesting that the performance gap between S/BP and S/BP-EtOH was not related with their initial different phases (Fig. 2c). The crystallite size of sulfur in S/BP was ~ 46.9 nm estimated by the Sherrer equation, which was comparable with that of S/BP-EtOH (~ 40 nm). Therefore, the crystallite size should not be the cause of the performance difference, neither. The superior performance of S/BP as compared with S/BP-EtOH can be attributed to the more homogeneous distribution of sulfur within S/BP as shown in Fig. 1f. This result contradicts with the previous study by Kim et al., where β-sulfur was claimed to show an improved performance after it was converted from α-sulfur by heat treatment [8]. However, their conclusion may be not that convincing since the S/C without heat treatment (α-sulfur) completely lost the macroscopic and microscopic ordered aspect and showed a much lower capacity than the average performance of α-sulfur in the literature [25–27]. It had also been demonstrated by both ex situ and in situ XRD in previous studies that orthorhombic α-sulfur was spontaneously converted to monoclinic β-sulfur after operation in LSBs [14, 26, 27] It is well known that α-sulfur is thermodynamically stable at ambient conditions while β-sulfur is uncommon in nature because it is only stable above 95.3 °C, and below this it readily converts to α-sulfur. It seems that the presence of a nonpolar carbon (after treatment with ethanol) could stabilize β-sulfur at ambient conditions.

The CV of S/BP as the cathode in LSB shows two cathodic peaks centered at 2.00 and 2.28 V and the corresponding anodic peaks located at 2.62 and 2.89 V, respectively (Fig. 2d). These are the characteristic peaks of sulfur de-/lithiation [17, 21, 23]. The cathodic peak at 2.28 V is associated with the formation of long-chain polysulfides from sulfur reduction, and the cathodic peak at 2.00 V is attributed to the subsequent transformation process of long-chain polysulfides to short-chain polysulfides (Li_2_S_2_/Li_2_S). The corresponding anodic peaks are related with the reverse conversion of short-chain polysulfides to sulfur. The CV of S/BP-EtOH is similar to that of S/BP.

### Electrocatalysis of Co_x_S Toward Polysulfide Conversion

**Fig. 3:**
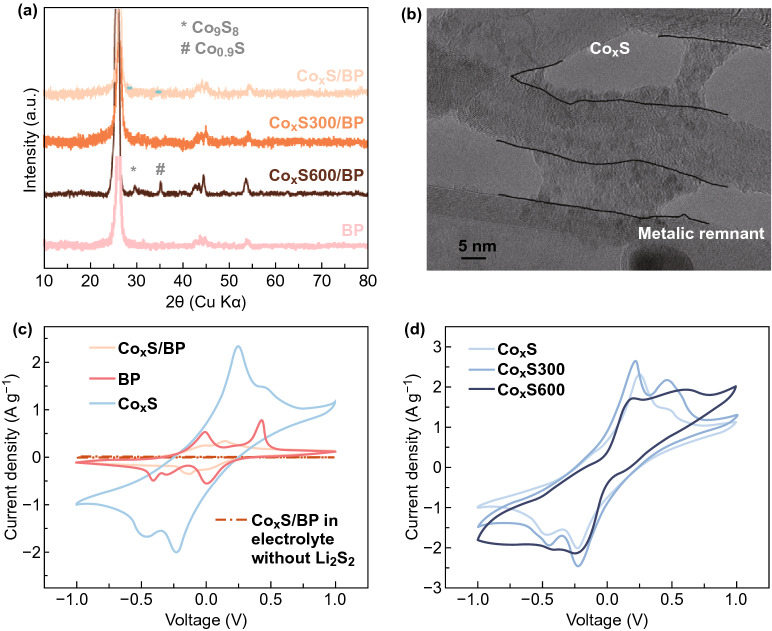
**a** XRD patterns of Co_x_S/BP, BP, Co_x_S300/BP, and Co_x_S600/BP. **b** TEM image of Co_x_S/BP. **c** CV of Co_x_S/BP electrode in symmetric cell containing electrolytes with/without 0.1 M Li_2_S_6_ at 3 mV s^−1^ in comparison with its constituents. **d** CV of symmetric cells using Co_x_S electrodes annealed by different temperatures

To further improve the cathode performance, Co_x_S catalyst on buckypaper (Co_x_S/BP) was prepared by electrodeposition in isopropanol solution containing thiourea and cobalt nitrate. Both the XRD patterns of Co_x_S/BP and Co_x_S300/BP show only peaks of BP, suggesting the possible lack of long-range ordered structure for Co_x_S and Co_x_S300 (Fig. 3a). The amorphous structure of electrodeposited Co_x_S has been reported by previous studies [28, 29]. When the annealing temperature was increased to 600 °C, Co_x_S600/BP demonstrated two additional peaks at 2*θ* = 29.6° and 2*θ* = 35.1°, which might be assigned to the peaks of Co_1-x_S. TEM image shows that Co_x_S nanoparticles were deposited on the surface of carbon nanotubes (CNTs) and aggregated (Fig. 3b). The aggregation of Co_x_S particles should be kinetically preferred because of the poor affinity of non-polar CNTs toward polar Co_x_S [13]. While CNTs showed clear lattice fringes, no clear lattice fringes were observed for Co_x_S, confirming amorphous structure of electrodeposited Co_x_S.

The catalytic activity of Co_x_S/BP toward the polysulfide conversion was examined by CV in symmetric cells using two identical electrodes. There are two pairs of redox peaks shown in the CV of Co_x_S/BP (Fig. 3c) with the anodic peaks centered at 0.15 and − 0.02 V, and the corresponding cathodic peaks are located at −0.01 and −0.15 V. No peaks were detected for the CV of Co_x_S/BP in the electrolyte without Li_2_S_6_. Surprisingly, the experiment control using BP as a substrate also shows two pairs of redox peaks (anodic peaks of 0.43 and − 0.01 V and the corresponding cathodic peaks of 0.001 and − 0.41 V) in its CV. Its catalytic activity for the polysulfide conversion might come from the metallic remnant (such as Fe) used for CNT synthesis. Because both Pt nanoparticles and Co nanoparticles had been reported to be catalysis active for polysulfide conversion and polysulfide conversion on non-polar carbonaceous materials were quite sluggish [19, 20, 22]. To rule out the catalytic effect of BP, Co_x_S was electrodeposited on the catalytic-inactive SS mesh. The deposited Co_x_S exhibited high reversibility with two pairs of redox peaks with the anodic peaks at 0.47 and 0.25 V and the corresponding cathodic peaks at − 0.23 and − 0.46 V. Since Li_2_S_6_ was the starting material in the cell, the redox peak of -0.46/0.25 V should be the conversion between Li_2_S_6_ and Li_2_S while the redox peak of −0.23/0.47 V should be the transformation between Li_2_S_6_ and S_8_ [20]. The annealing temperature effect on the polysulfide catalysis of BP and Co_x_S was also studied. BP showed no much difference on CV curves after annealed at different temperatures (Fig. S3). While the CV of Co_x_S300 was similar to that of Co_x_S, Co_x_S600 only showed a pair of broad redox peaks (Fig. 3d), suggesting that too high annealing temperature was detrimental to the catalysis. This might be due to the loss of the structure defects acting as active sites after high-temperature annealing.

**Fig. 4:**
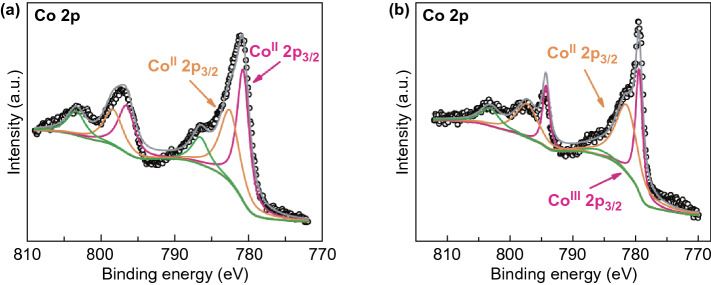
**a** Co 2p spectra of Co_x_S before polysulfide catalysis in a symmetric cell. **b** Co 2p spectra of Co_x_S after polysulfide catalysis in a symmetric cell

XPS was used to analyze the surface chemistry of Co_x_S before and after catalysis. The quantification analysis of original Co_x_S (Fig. 4a) suggests an atomic ratio of ~ 2:1 for Co/S, indicating its hybrid composition containing additional component of cobalt oxide/hydroxide [28, 29]. The Co 2p_3/2_ peak of the original Co_x_S can be deconvoluted into two peaks located at 780.7 and 782.5 eV, corresponding to their Co 2p_1/2_ peaks of 796.5 and 798.3 eV, respectively. Both band separations between the Co 2p_3/2_ peak and its corresponding Co 2p_1/2_ peak are ca. 15.8 eV, suggesting that the cobalt cations are in Co(II) valence state for the hybrid. The Co 2p_3/2_ peaks at 780.7 and 782.5 eV can be assigned to the presence of Co^II^_x_S and Co^II^(OH)_2_, respectively [30, 31]. After catalyzing the polysulfide conversion, the Co 2p_3/2_ peak of Co_x_S (Fig. 4b) can be deconvoluted into two peaks located at 779.4 and 781.4 eV with the corresponding Co 2p_1/2_ peaks at 794.2 and 797.2 eV, respectively. The Co 2p_3/2_ peak of 779.4 eV and the band separation of 14.8 eV with its corresponding Co 2p_1/2_ peak can be assigned to Co(III) valence state, indicating the partial oxidation of Co(II) to Co(III) and the significant role of Co(II)/Co(III) redox in the catalysis [32]. It is believed that Co(II) content is dominant in Co_x_S during discharge, acting as electron donor to assist the reduction of polysulfide. Itself is oxidized to be Co(III) but is converted to Co(II) again by accepting electrons from cathode. Similarly, Co(III) content is dominant in Co_x_S during charge, acting as electron acceptor to oxidize polysulfide. S 2p XPS spectra after catalysis show peaks of the terminal sulfur (S_T_^−1^, 162.7 eV), bridging sulfur (S_B_^0^, 163.9 eV) atoms and polythionate (168.9 eV), respectively (Fig. S3b) [33]. This provides more evidence on the S–S from S_8_ or longer-chain polysulfide to yield polythionate complex with the assistance of Co(II)/Co(III) redox. The signal ratio between S_T_^−1^ and S_B_^0^ is ca. 5.7:1, indicating the dominant presence of the former. This is due to the terminal voltage of the symmetric cell at 0 V (reversed from 1 V), resulting in Li_2_S_2_ or Li_2_S as the major material on the Co_x_S surface. The discharge scenario may be described as follows: solid S_8_ is initially reduced to form liquid Li_2_S_8_, which transfers and adsorbs on catalyst surface due to polar–polar reaction. Soluble Li_2_S_8_ is then reduced to form high-order polysulfides (Li_2_S_x_, 4 ≤ x ≤ 7) and finally to form Li_2_S_2_ or Li_2_S by accepting electrons from Co(II) species twice. Co(II) is oxidized to be Co(III) and is converted to Co(II) again by accepting electrons from cathode. Further studies on the detailed catalytic mechanism of Co(II)/Co(III) redox species toward polysulfide conversion are highly interesting and important in improving the catalysis in LSBs.

### Electrodeposition of Sulfur on Co_x_S-decorated BP (S/Co_x_S/BP)

**Fig. 5:**
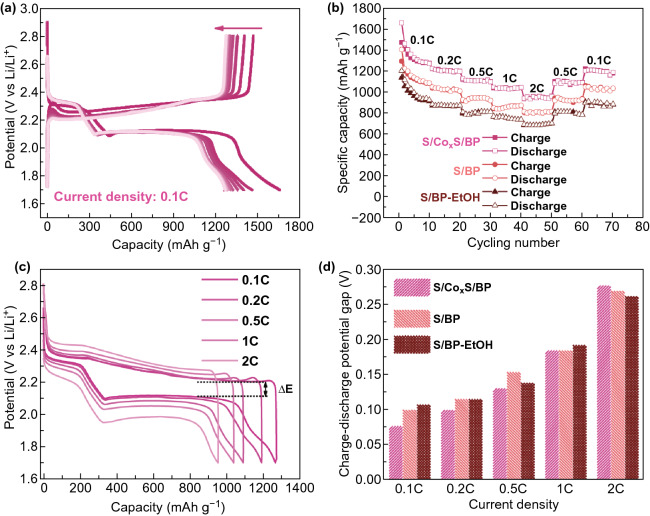
**a** Discharge–charge profile of S/Co_x_S/BP at the rate of 0.1 C. **b** Rate capability of S/Co_x_S/BP compared with S/BP and S/BP-EtOH. **c** Discharge–charge profile of S/Co_x_S/BP at the rate from 0.1 to 2 C. **d** Derived discharge–charge potential gap along with the current density

The activity of S/Co_x_S/BP was also evaluated in a LSB with Co_x_S as the catalyst to further improve the performance. The capacity contribution by Co_x_S is negligible since its lithiation voltage is below 1.5 V vs. Li/Li^+^ while the cutoff voltage is 1.7 V in this work [20]. The discharge capacity was as high as 1650 mAh g^−1^ at the first cycle, which was close to the theoretical value of sulfur cathode capacity (Fig. 5a). The rate capability of S/Co_x_S/BP is compared with S/BP and S/BP-EtOH at step current rates of 0.1, 0.2, 0.5, 1 and 2 C (Fig. 5b). All of them show decreased capacities along with the increase in current rate and good capacity retention when the current rate is reverted to 0.5 and 0.1 C. Among them, S/Co_x_S/BP demonstrates the highest capacities of 1280, 1190, 1100, 1030 and 950 mAh g^−1^ at 0.1, 0.2, 0.5, 1 and 2 C, respectively, alluding to the advantage of Co_x_S promoting sulfur performance by catalyzing polysulfide conversion and efficiently mitigating the shuttle effect of polysulfide. The performance order is S/Co_x_S/BP > S/Co_x_S300/BP > S/Co_x_S600/BP at step current rates, suggesting that annealing might result in the loss of defects and as such reduce the activity of Co_x_S catalyst (Fig. S5a).

Metal sulfide can act as a polar host material of polysulfides to effectively reduce the shuttle effect via the polar–polar adsorption and thus contribute to improve the battery performances. To distinguish the polar adsorption from the catalytic effect, the visual adsorption test is used to evaluate the adsorption capability of the catalyst. It is expected that BP showed no apparent effect on the polysulfide adsorption because of the non-polar property (Fig. S3c). However, while Co_x_S300 shows quite good adsorption capability to decolorize the Li_2_S_6_ solution, Co_x_S has weak adsorption capability, suggesting that the performance improvement in S/Co_x_S/BP can be attributed to the enhanced polysulfide conversion by the catalysis effect of Co_x_S instead of the polar–polar adsorption. Cathodes with different ratios from 5:1 to 7:1 between sulfur and catalyst were fabricated to optimize the catalytic efficiency (Fig. S3d). S/Co_x_S/BP in ratio of 6:1 shows the highest capacity among them. This indicates that higher catalyst content may deteriorate electron transfer between the active material and BP, although it can provide more active catalytic sites for polysulfide conversion. Thus, the ratio of 6:1 between sulfur and catalyst has the optimal catalytic effect.

The voltage gap (∆*E*) of the charge–discharge plateau can be used to evaluate the efficiency of LSB. The lower the voltage gap, the higher the efficiency. S/Co_x_S/BP showed the ∆*E* of 77, 100, 131, 184 and 277 mV at the current rate of 0.1, 0.2, 0.5, 1 and 2 C, respectively (Fig. 5c). These values were lower than those of S/BP and S/BP-EtOH at current rate below 1 C, suggesting the better efficiency of S/Co_x_S/BP (Fig. 5d). However, the advantage of Co_x_S on ∆*E* reduction gradually decreased along with the increase in current rate and finally disappeared at a current rate of 2 C. Similar phenomenon was also observed by Jiang et al. [21]. This should be due to the insulating properties of sulfur and its discharge products, resulting in the increase in the voltage loss along with the increase in current rate. Such voltage loss from the internal resistance becomes a dominant component of the ∆*E* at high current rate and thus offsets the reduction in potential gap by Co_x_S.

**Fig. 6:**
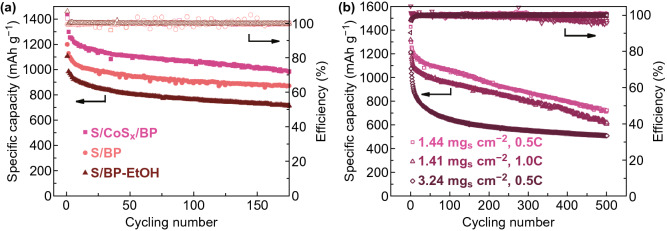
**a** Cycling performance of S/Co_x_S/BP, S/BP and S/BP-EtOH at 0.5 C. **b** Continuous operation of S/Co_x_S/BP up to 500 cycles

The cycling performance was carried out typically at 0.5 C rate for S/Co_x_S/BP, S/BP and S/BP-EtOH. S/Co_x_S/BP shows a high initial discharge capacity of 1420 mAh g^−1^, which maintains as high as 1000 mAh g^−1^ after 170 cycles while S/BP and S/BP-EtOH exhibit both lower initial discharge capacities and more severe capacity fading (Fig. 6a). Similarly, the cycling performance of S/Co_x_S/BP is also superior to both S/Co_x_S300/BP and S/Co_x_S600/BP (Fig. S4a). After 500 cycles of continuous operation, S/Co_x_S/BP still possesses the capacity of 715 mAh g^−1^ with a fade rate of 0.099% per cycle and the high efficiency of ~ 100% (Fig. 6b). The good performance can be kept when the rate is increased from 0.5 to 1.0 C. The high performance of S/Co_x_S/BP could be attributed not only to the good dispersion of the electrodeposited nanostructured sulfur within SDS treated BP, but also from the presence of Co_x_S catalyst promoting the kinetic of the polysulfide conversion. The good cycling stability of S/Co_x_S/BP also benefits from such catalytic effect of Co_x_S, which suppresses the shuttle effect by reducing the accumulation of polysulfide species at the cathode. However, when the S loading is doubled, the performance degradation rate is significantly increased, possibly due to the sulfur aggregation on the near-surface area without the utilization of BP bulk area. Compared to other studies on electrodeposited sulfur used for the LSB, the S/Co_x_S/BP cathode of this work shows the best performance (Table S1). Even in comparison with previous studies of catalysts used in LSBs, S/Co_x_S/BP cathode performs better and is clearly a promising choice because of its facile and fast preparation (Table S2).

## Conclusions

In the present work, a high-performance cathode for lithium–sulfur batteries based on sulfur active material electrodeposited on carbon nanotubes buckypaper was developed. Sulfur electrodeposition was accomplished by time- and energy-efficient procedure based on electrooxidation of polysulfides in water. A Co_x_S catalyst was also straightforwardly electrodeposited on buckypaper significantly increasing the performance of the electrode. The buckypaper was pretreated by SDS to form a polar sulfate layer on the carbon nanotubes to assist bonding of polysulfide and therefore improving the homogeneity of the electrochemically deposited sulfur. This improved significantly the performance of the system. Moreover, both the capacity and the cycling stability could be further improved by the presence of Co_x_S, which catalyzed the polysulfide conversion. The enhanced kinetics of the polysulfide conversion mitigated the accumulation of the polysulfide intermediates and suppressed their diffusion to the anode. In essence, the study demonstrates that electrodeposition offers significant advantages for the formation of high-performance cathode for lithium-sulfur battery.

## Electronic supplementary material

Below is the link to the electronic supplementary material.

Supplementary file1 (PDF 558 kb)
