# Supplementary material for: Electrodeposited Sulfur and CoxS Electrocatalyst on Buckypaper as High-Performance Cathode for Li–S Batteries
Source: Nanomicro Lett. 2020 Jul 3;12:141. doi: 10.1007/s40820-020-00479-1 (PMC7770841; doi:10.1007/s40820-020-00479-1)
Supplement: Supplementary file 1 — Supplementary file1 (PDF 558 kb) [file 40820_2020_479_MOESM1_ESM.pdf]

Supporting Information for

Electrodeposited Sulfur and Co_xS Electrocatalyst on Buckypaper as High-Performance Cathode for Li-S Batteries

Yi Zhan[1, 3, +], Andrea Buffa[2, +], Linghui Yu[1], Zhichuan J. Xu[1, 3, *], Daniel Mandler[2, 3, *]

[1]School of Materials Science and Engineering, Nanyang Technological University, 639798, Singapore

[2]Institute of Chemistry, The Hebrew University of Jerusalem, Jerusalem, 9190401, Israel[3]Singapore-HUJ Alliance for Research and Enterprise (SHARE), Campus for Research Excellence and Technological Enterprise (CREATE), 138602, Singapore

+Yi Zhan and Andrea Buffa contributed equally to this work

*Corresponding authors. E-mail: xuzc@ntu.edu.sg (Zhichuan J. Xu); daniel.mandler@mail.huji.ac.il (Daniel Mandler)

Supplementary Figures and Tables

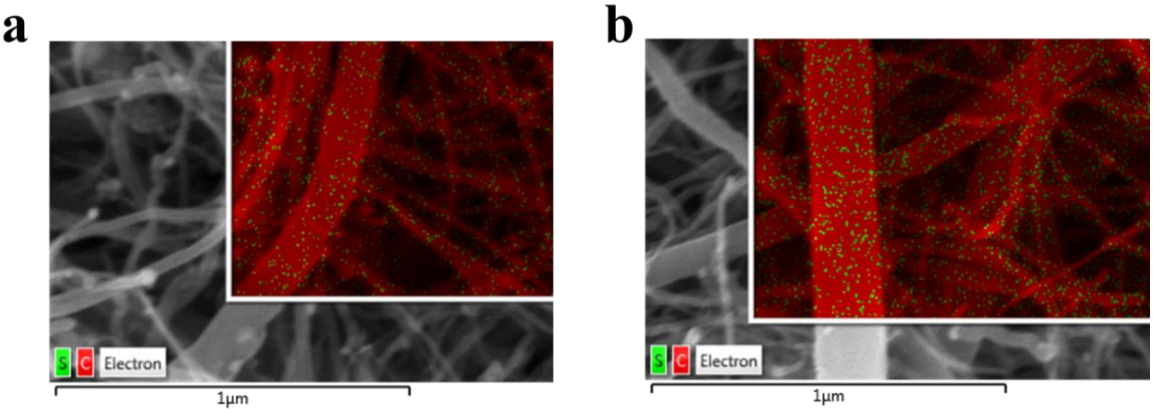

Fig. S1 Surface elemental mapping of S/BP (**a**) and S/BP-EtOH (**b**) (green for sulfur and red for carbon)

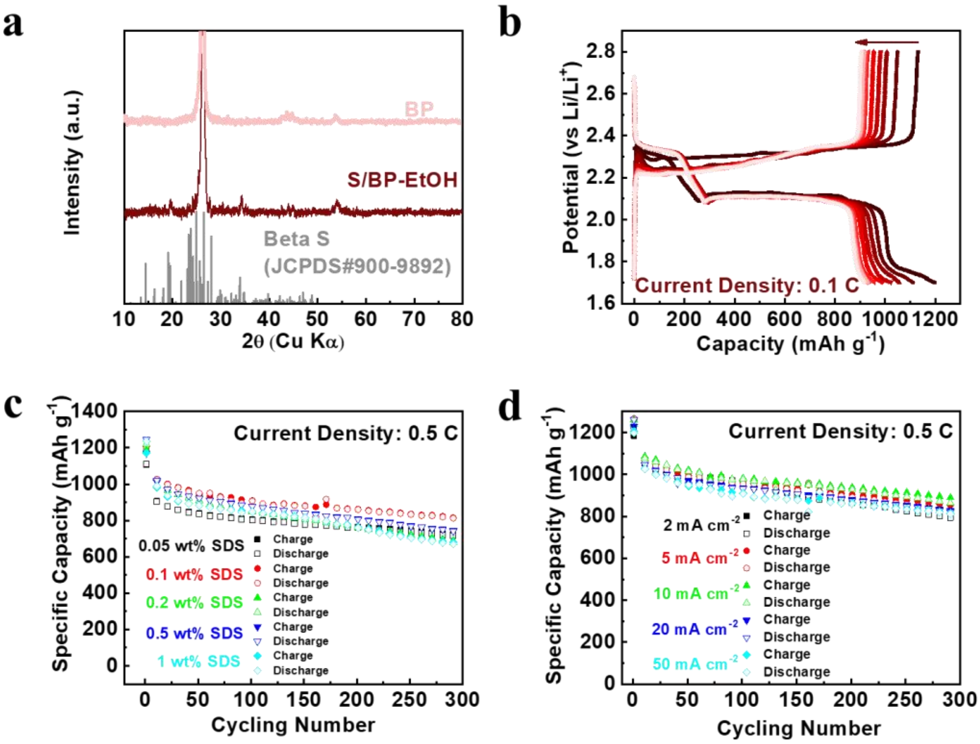

Fig. S2 (**a**) XRD pattern of S/BP-EtOH; (**b**) the discharge-charge profile of S/BP-EtOH at the rate of 0.1 C; (**c**) the cycling stability of S/BP pretreated with varied SDS concentration at a constant current density of 10 mA cm^{-2}; (**d**) and the cycling stability of S/BP prepared by different current densities upon treatment of 0.1 wt% SDS

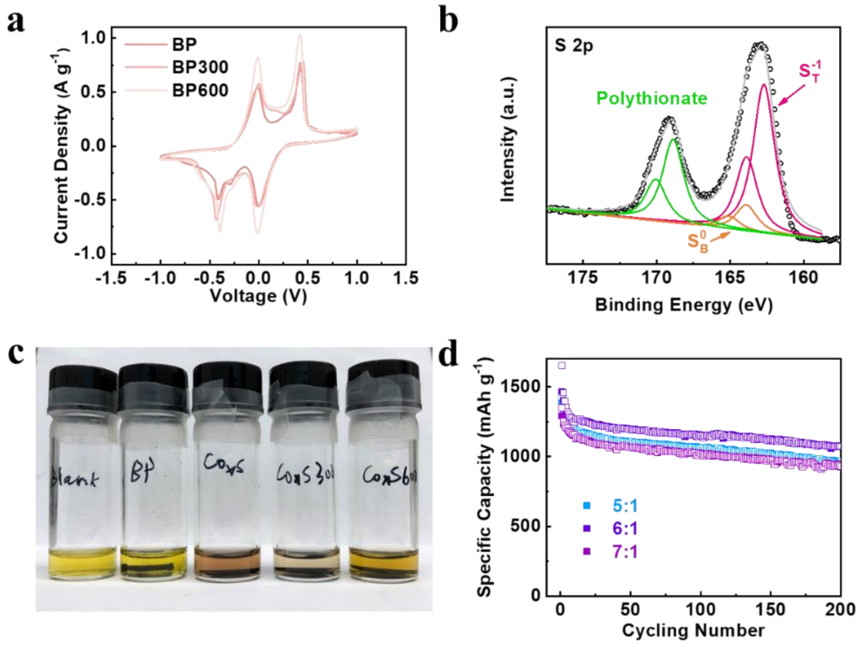

Fig. S3 (**a**) CV of symmetric cells using BP annealed by different temperature; (**b**) S 2p spectra of Co_xS after polysulfide catalysis in a symmetric cell; (**c**) adsorption test of different samples (from left to right: bare Li_2S_6 solution, BP, Co_xS/SS, Co_xS300/SS and Co_xS600/SS); (**d**) charge-discharge performance of S/Co_xS/BP with different mass ratio of S:Co_xS from 5:1 to 7:1 at 0.5 C

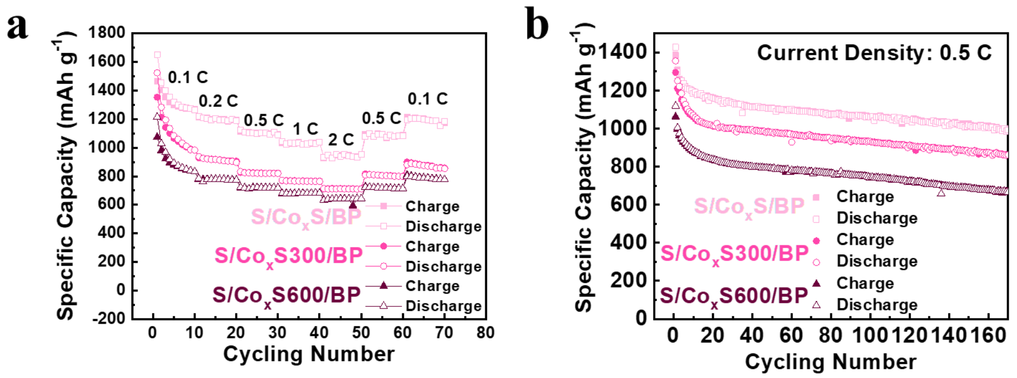

Fig. S4 (**a**) The rate capability of S/Co_xS/BP as compared with S/Co_xS300/BP and S/Co_xS600/BP (**b**) Their cycling performance at 0.5 C

Table S1 Performance comparison of sulfur cathodes prepared by electrodeposition

Cathodes	Rate performance (mAh g^{-1})	Initial capacity (mAh g^{-1})	Ending capacity (mAh g^{-1})	References
S/Co_xS/BP	940 (2 C)	1427 (0.5C)	715 (0.5C, 500 cycles)	This work
Sulfur in the monolithic carbon	725 (2C)	1068 (0.5C)	612 (0.5C, 500 cycles)	ACS Nano 2016, 10, 1633
Graphene-sulfur composites	625 (2 A g^{-1})	400 (5 A g^{-1})	260 (5 A g^{-1}, 200 cycles)	J. Mater. Chem. A,2015, 3,16513
Sulfur on nickel foam	786 (2 C)	1135 (0.5C)	895 (0.5C, 300 cycles)	Nano Lett. 2015, 15, 721
Mesoporous carbon–sulfur composite	590 (2 A g^{-1})	860 (0.5 A g^{-1})	857 (0.5 A g^{-1}, 200 cycles)	J. Mater. Chem. A, 2017, 5, 5905

Table S2 Performance comparison of sulfur cathodes assisted by the catalysts

Cathodes	Rate performance (mAh g^{-1})	Initial capacity (mAh g^{-1})	Ending capacity (mAh g^{-1})	References
Electrodeposited Co_xS nanocrystals	940 (2C)	1427 (0.5C)	715 (0.5C, 500 cycles)	This work
VO_2(P) nanoparticles	760 (2C)	880 (2C)	780 (2C, 500 cycles)	Nano Energy 57 (2019) 230
Pd nanoparticles	578 (2C)	805 (1C)	587 (1C, 400 cycles)	Carbon 143 (2019) 878
$NiFe_2O_4$ nanopar ticles	734 (2C)	800 (1C)	600 (1C, 1000 cycles)	Carbon 142 (2019) 32

Black phosphorus quantum dots	821 (2C)	1234 (0.1C)	1072 (0.1C, 200 cycles)	Nat. Comm. (2018) 9, 4164
Boron-carbide nanowires	~700 (2C)	1024 (1C)	815 (1C, 500 cycles)	Adv. Mater. 2018, 30, 1804149
ZnS_2 nanospheres	876 (2C)	1187 (0.2C)	896 (0.2C, 200 cycles)	Nano Energy 51 (2018) 73
Co-doped VN yolk–shell nanospheres	650 (2C)	1050 (0.5C)	715 (0.5C, 200 cycles)	ACS Appl. Mater. Interfaces 2018, 10, 11642
MoP_2 nanoparticles	521 (2C)	619 (0.1C)	543 (0.1C, 500 cycles)	Small 2018, 14, 1702853
Co_3S_4 nanotube	750 (2C)	975 (0.5C)	~800 (0.5C, 200 cycles)	Nano Energy 37 (2017) 7
ReS_2 nanosheets	787 (2C)	920 (0.5C)	750 (0.5C, 300 cycles)	Nano Lett. 2016, 16, 3780
MoS_{2-x} nanoflakes	1000 (2C)	1160 (0.5C)	628 (0.5C, 600 cycles)	Energy Environ. Sci., 2017, 10, 1476
Phosphorene nanosheets	865 (2C)	850 (1C)	660 (1C, 500 cycles)	Adv. Mater. 2017, 29, 1602734
Co nanoparticles	534 (2C)	993 (0.5C)	607 (0.5C, 500 cycles)	Adv. Energy Mater. 2017, 7, 1602543
MnO_2 nanosheets	890 (2C)	1100 (0.5C)	840 (0.5C, 200 cycles)	Nat. Comm. (2015) 6, 5682
Fe_2O_3 nanoparticles	705 (2C)	700 (2C)	380 (2C, 500 cycles)	Nano Energy 33 (2017) 306
CoS_2	1003 (2C)	1368 (0.5C)	1005 (0.5C, 150 cycles)	Nano Lett. 2016, 16, 519
VS_2	700 (2C)	830 (0.5C)	701 (0.5C, 300 cycles)	PNAS 2017, 114 (5), 840
VN nanoribbons	956 (2C)	1128 (1C)	917 (1C, 200 cycles)	Nat. Comm. (2017) 8, 14627
TiN	579 (2C)	1069 (0.2C)	748 (0.2C, 50 cycles)	ACS Energy Lett. 2017, 2, 327
TiO_2-TiN nanoparticles	682 (2C)	1008 (0.3C)	927 (0.3C, 300 cycles)	Energy Environ. Sci., 2017, 10, 1694
